# Limited Wegener's granulomatosis presenting as lung nodules in a patient with rheumatoid arthritis: a case report

**DOI:** 10.1186/1757-1626-1-417

**Published:** 2008-12-23

**Authors:** Sushma Pai, Mukta Panda

**Affiliations:** 1 Department of Medicine, University of Tennessee, Chattanooga, Tennessee, USA

## Abstract

**Background:**

Rheumatoid arthritis has varied pleuroparenchymal manifestations. Wegener's granulomatosis can develop in an established case of rheumatoid arthritis and this association although previously reported is very rare.

**Case presentation:**

A 60-year-old lady had been diagnosed with rheumatoid arthritis on the basis of her clinical symptoms and serological tests which were positive RA factor and anti-CCP antibodies. Her rheumatoid arthritis activity had been mild and well controlled with hydroxychloroquine and low dose prednisone. She presented with a productive cough and right-sided pleuritic chest pain. CT scan of the chest showed three lung nodules with increased uptake on PET CT scan, raising concerns for an inflammatory or malignant process. The differential diagnosis included rheumatoid nodules, infections or malignancy. A CT-guided needle biopsy of the largest nodule was undertaken which showed vasculitis typical of Wegener's granulomatosis. Stains and cultures of the biopsy specimen were negative for bacteria, fungi and acid fast bacilli. A panel of serological tests for vasculitis were checked and showed elevated titers of cANCA and anti-proteinase 3 antibodies. Urine analysis and CT scan of paranasal sinuses was normal. Since the upper respiratory tract and the kidneys were spared a diagnosis of limited Wegener's granulomatosis affecting only the lungs was made. Due to the toxicity of cyclophosphamide, her relatively mild disease sparing the kidneys and the underlying rheumatoid arthritis, weekly methotrexate was started and low dose prednisone was continued. She had marked symptomatic improvement and complete resolution of the nodules was documented on subsequent imaging.

**Conclusion:**

Wegener's granulomatosis developing in a patient with rheumatoid arthritis is very rare but should be considered as it warrants a different and possibly more aggressive treatment approach.

## Introduction

Rheumatoid arthritis (RA) is a systemic inflammatory disorder that has pleuroparenchymal involvement with varied manifestations, which includes organizing pneumonia, interstitial fibrosis, rheumatoid nodules, airway disorders such as bronchiectasis and bronchiolitis and pulmonary vasculitis. Although the development of Wegener's granulomatosis (WG) in a patient with pre-existing RA is rare, it can occur as autoimmunity is the basis of collagen vascular diseases. We present a rare case of limited Wegener's granulomatosis presenting only as lung nodules in a patient with RA.

## Case presentation

A 60-year-old lady presented with a one-week history of shortness of breath, low-grade fever and right-sided pleuritic chest pain. She had been treated with azithromycin for presumed bronchitis with no improvement. She had RA factor positive rheumatoid arthritis diagnosed about 3 years prior and was doing well with hydroxychloroquine and low dose prednisone. Her other medical problems included osteopenia, gastroesophageal reflux disease and hypercholesterolemia for which she was on weekly risedronate, calcium and vitamin D supplements, omeprazole and atorvastatin respectively.

On examination her vital signs were stable, she was afebrile and her oxygen saturation was 95% on room air. She appeared tired but not in any distress. Auscultation of the lungs revealed wheezes bilaterally and few crackles at the right base. The remainder of her examination was unremarkable. She did not have any synovitis in the joints at this time and range of motion was normal in all joints with no muscle wasting. Skin examination showed some old hypopigmented macules over her neck and face. There were no skin nodules.

**Figure 1:**
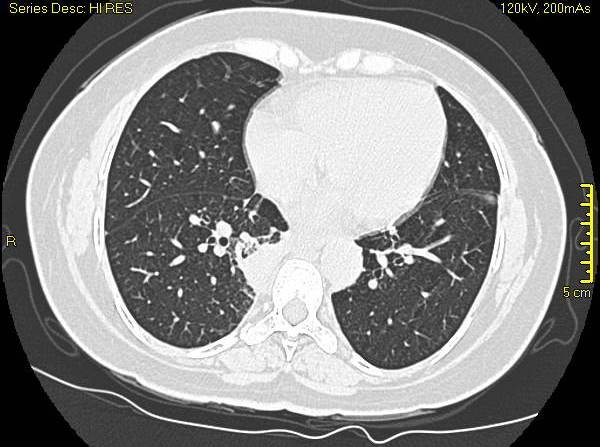
**High resolution CT scan of the chest showing the lung nodules**.

**Figure 2:**
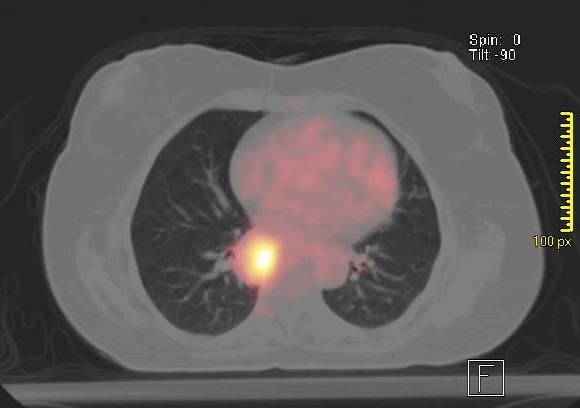
**PET CT scan of chest showing increased uptake in nodules**.

**Figure 3:**
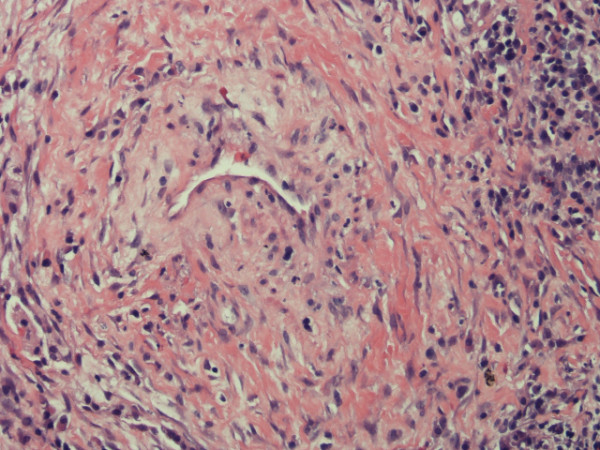
**Histopathology of lung biopsy showing vasculitis**.

Her white count was 7.8 × 10^9^/l with neutrophils of 84%. The ESR by Westergren's method was 46 mm at the end of one hour. A comprehensive metabolic profile was normal and urine analysis did not show any sediment. Her chest radiograph showed streaky infiltrates at the right base. A high resolution CT scan showed three lung nodules (Fig 1). An FDG PET scan was done which showed avid uptake in all the nodules with an SUV value of 8 (Fig 2). A CT-guided lung biopsy of the right lung nodule was performed. The lung biopsy showed fibrosis with acute and chronic inflammation and necrotizing vasculitis (Fig 3). Biopsy specimen examined for aerobic and anaerobic bacteria, acid fast bacilli and fungus, was negative on smear and culture. A complete vasculitis panel was obtained which included antinuclear antibodies, anti-dsDNA, anti-Smith, anti-SSA and SSB, anti-histone antibody, anti-Jo-1, anti-centromere antibody, anti Scleroderma -70, anti-proteinase 3 and anti-myeloperoxidase antibodies along with the antineutrophil cytoplasmic antibodies (ANCA). The only positive tests were an elevated cANCA in a titer of 1: 640 (Normal less than 1: 20) and anti-proteinase 3 antibodies of 118 units (Normal 0 – 20 units).

A screening CT scan of the sinuses was normal. A two-dimensional echocardiogram showed a left ventricular ejection fraction of 55% and normal pulmonary artery pressures. Based on the biopsy and the serological findings, a diagnosis of limited Wegener's granulomatosis presenting as lung nodules was made.

Methotrexate was started and the low-dose prednisone was continued. Her symptoms improved dramatically with these and a repeat CT scan of the chest obtained one month after treatment showed regression in the size of the nodules and one done 3 months later showed complete resolution of the same. She will be followed closely for development of kidney or upper respiratory tract involvement and monitored for methotrexate toxicity.

## Discussion

The differential diagnosis of lung nodules in this patient would include bronchiolitis obliterans organizing pneumonia, rheumatoid nodules, malignancy including lymphoma, and infections such as tuberculosis. The necrotizing vasculitis in the biopsy specimens and the positive cANCA indicated WG co-existent with the RA. The presence of RA is confirmed by the erosive arthritis in the joints of her hands and positive RA and anti CCP antibodies at the time of diagnosis.

She developed WG in addition to her preexisting RA, which is very rare, with only several cases reported so far [1-4]. In the reported cases the majority were women and RA preceded WG in all cases except one.

Based on the anatomic ELK classification [5] (E for ear, nose and throat, L for lung and K for kidney) of Wegener's granulomatosis, she appeared to have the L type with lung involvement only with sparing of the kidney and the upper respiratory tract. Lung involvement in Wegener's granulomatosis may manifest as lung nodules, which are often cavitary, acinar shadows which may represent alveolar hemorrhage, mediastinal lymphadenopathy and pleural effusions. Traditionally the treatment of WG has been cyclophosphamide-based regimens; however these are limited by the toxicity of this drug, which may manifest as bone marrow suppression, infections, cystitis, myelodysplasia and bladder cancer [6]. The risk of cancer directly depends on the cumulative dose of cyclophosphamide. The European Vasculitis Study Group [7] compared the use of azathioprine after achieving remission with cyclophosphamide versus treatment with cyclophosphamide for the entire duration of treatment and they found that the rate of relapse did not differ significantly in the two groups who were followed up to 18 months. Thus the duration of treatment with cyclophosphamide can be safely reduced. In non life threatening WG or when critical organs are not involved, methotrexate may be used to achieve remission of the disease but the relapse rate is higher which may necessitate longer than 12 months of treatment [6,8]. The lung being one of the critical organs, if our patient had presented with pulmonary alveolar hemorrhage, cyclophophamide pulses would have been appropriate. But lung nodules being a relatively benign manifestation of WG, cyclophosphamide may be used initially to induce a remission after which azathioprine, methotrexate or leflunomide may be used as maintenance therapy to prevent relapses. We chose methotrexate for our patient and continued low dose prednisone that she tolerated well. Methotrexate would likely also benefit in controlling the activity of her RA as well.

Although she has limited WG, there are reports of ominous complications such as the development of complete heart block [9] and hence she will be closely monitored for those as well as for progression to the more severe form involving the kidneys.

## Conclusion

WG developing in patients with RA although very rare is possible and should be considered when necrotizing vasculitis and cANCA positivity is seen. WG that does not involve the kidneys or other vital organs can be treated with less toxic regimes with methotrexate or azathioprine. Limited WG may progress to involve kidneys and other organs and hence should be clinically monitored. As autoimmune dysfunction is the postulated mechanism in connective tissue disorders, these may coexist in the same patient. Thus clinicians should have a low threshold for keeping these in their differential diagnosis.

## Consent

Written informed consent was obtained from the patient for publication of this case report and accompanying images. A copy of the written consent is available for review by the Editor-in-Chief of this journal.

## Competing interests

The authors declare that they have no competing interests.

## Authors' contributions

SP was the resident involved in the care of this patient, and was responsible for preparing the manuscript. MP is the senior attending physician directing this patient's care and she edited the manuscript, critically reviewed it and made changes as necessary. Both the authors have read and approve of the final version.
